# Serum TRPM1 Autoantibodies from Melanoma Associated Retinopathy Patients Enter Retinal ON-Bipolar Cells and Attenuate the Electroretinogram in Mice

**DOI:** 10.1371/journal.pone.0069506

**Published:** 2013-08-01

**Authors:** Wei-Hong Xiong, Robert M. Duvoisin, Grazyna Adamus, Brett G. Jeffrey, Celia Gellman, Catherine W. Morgans

**Affiliations:** 1 Casey Eye Institute, Oregon Health & Science University, Portland, Oregon, United States of America; 2 Department of Physiology & Pharmacology, Oregon Health & Science University, Portland, Oregon, United States of America; 3 National Eye Institute, National Institutes of Health, Bethesda, Maryland, United States of America; The University of Melbourne, Australia

## Abstract

Melanoma-associated retinopathy (MAR) is a paraneoplastic syndrome associated with cutaneous malignant melanoma and the presence of autoantibodies that label neurons in the inner retina. The visual symptoms and electroretinogram (ERG) phenotype characteristic of MAR resemble the congenital visual disease caused by mutations in TRPM1, a cation channel expressed by both melanocytes and retinal bipolar cells. Four serum samples from MAR patients were identified as TRPM1 immunoreactive by 1. Labeling of ON-bipolar cells in TRPM1+/+ but not TRPM1−/− mouse retina, 2. Labeling of TRPM1-transfected CHO cells; and 3. Attenuation of the ERG b-wave following intravitreal injection of TRPM1-positive MAR IgG into wild-type mouse eyes, and the appearance of the IgG in the retinal bipolar cells at the conclusion of the experiment. Furthermore, the epitope targeted by the MAR autoantibodies was localized within the amino-terminal cytoplasmic domain of TRPM1. Incubation of live retinal neurons with TRPM1-positive MAR serum resulted in the selective accumulation of IgG in ON-bipolar cells from TRPM1+/+ mice, but not TRPM1−/− mice, suggesting that the visual deficits in MAR are caused by the uptake of TRPM1 autoantibodies into ON-bipolar cells, where they bind to an intracellular epitope of the channel and reduce the ON-bipolar cell response to light.

## Introduction

Melanoma associated retinopathy (MAR) is a paraneoplastic syndrome in some patients with cutaneous malignant melanoma characterized by the presence of serum autoantibodies against retinal proteins [1]–[9] and by visual deficits including: flickering photopsias, night blindness, and a generalized constriction of visual fields. Electroretinogram (ERG) recordings from MAR patients show a “negative” ERG in which the b-wave, originating from the depolarization of ON-bipolar cells, is more severely affected than the a-wave, originating from the light-induced hyperpolarization of photoreceptors [1], [2], [9], [10]. Serum from MAR patients contains autoantibodies that label retinal bipolar cells [3], [4]. Intravitreal injection of purified IgG from MAR patients into monkey eyes reduced the amplitude of the ERG b-wave, indicating that MAR IgG has a reactive component affecting retinal function and suggesting that the vision abnormalities experienced by MAR patients result from autoantibodies [11].

An important breakthrough in elucidating the signal transduction pathway of retinal ON-bipolar cells was the identification of TRPM1 as the mGluR6-coupled ion channel [12]–[14]. TRPM1 is co-localized with mGluR6 at the tips of ON-BPC dendrites where they receive input from photoreceptors and, like mGluR6, has since been found to be a major locus of mutations causing complete congenital stationary night blindness (CSNB1) in humans [15]–[18]. The experiences of night blindness and the ERG b-wave reduction of MAR patients is also typical of CSNB1 [19]. Significantly, the other known site of TRPM1 expression is melanocytes [20].

Thus we proposed that autoantibodies in MAR patients' sera may bind TRPM1 cation channels in bipolar cells and inhibit the light response of the cell [21]. Recently, two reports from other groups [22], [23] have shown that indeed MAR patient sera contain autoantibodies against TRPM1.

Here, we report that TRPM1 autoantibodies from MAR patient sera bind to an epitope in the intracellular domain of the TRPM1 channel. They are internalized by live bipolar cells, and can reduce the b-wave of ERG from mouse eyes after intravitreal injection of IgG.

## Materials and Methods

### Patient Sera

Patient sera were acquired through the Ocular Immunology Laboratory, Oregon Health and Science University (OHSU). The serum samples are previously collected, tissue banked samples that are de-identified using code numbers rather than patient names, therefore patient consent for this study was not sought. Serum samples selected for this study were from patients with cutaneous malignant melanoma and visual deficits consistent with MAR, and which labeled bipolar cells in retina sections from mouse and macaque (not shown). The study has been approved by the OHSU Institutional Review Board. Serum sample # 2 in this study has been shown to react with recombinant TRPM1 on western blots [23].

### Animals

Adult C57BL6 mice, as well as TRPM1−/− mice (TRPM1^tm1Lex^; Texas Institute of Genomic Medicine, College Station, TX) and TRPM1+/+ littermates were maintained and used in accordance with guidelines provided by the NIH. All animal procedures were approved by the OHSU Institutional Animal Care and Use Committee.

### Cell culture and transfection

CHO-K1 cells were transfected with plasmids encoding EGFP-mTRPM1 (full-length mouse TRPM1 fused to the C-terminus of EGFP), or EGFP-huTRPM1 (full-length human TRPM1 fused to the C-terminus of EGFP) using TransIT-CHO Tranfection Kit (Mirus, Madison, WI) according to the manufacturer's instructions, then processed for immunofluorescence according to the protocol used for retina sections [13]. Transfected cells were incubated with a range of dilutions of MAR sera (1∶10 to 1∶1000), and the immunoreactivity visualized with anti-human IgG coupled to a red fluorophore (Alexa-594).

### Immunofluorescence

Mouse retina sections were prepared and processed for immunofluorescence confocal microscopy as previously described [13]. Primary antibodies and the dilutions used were: human MAR sera (1∶10 to 1∶1000), rabbit anti-TRPM1 antibody (1∶200; HPA014785, Sigma-Aldrich, St Louis, MO), mouse anti-PKC alpha (1∶5000; Novus Biologicals, Littleton, CO). Secondary antibodies used were: anti-rabbit IgG or anti-mouse IgG conjugated to Alexa Fluor 488, anti-human IgG conjugated to Alexa Fluor 594 (all used at 1∶1000; Invitrogen, Grand Island, NY). Fluorescence images of retina sections, transfected cells and dissociated retinal cells were acquired with an Olympus FluoView FV1000 confocal microscope using a 60×/1.42 oil immersion objective. Image brightness and contrast were enhanced using Adobe Photoshop (Adobe Systems Inc, San Jose, CA).

### Intravitreous injections

MAR IgG was purified from whole serum by binding to protein A/G agarose (Pierce Biotechnology Inc., Rockford IL) followed by elution with low pH into a neutralizing buffer. Intravitreal injections of active IgG or inactive IgG were performed to examine the effects of TRPM1-positive MAR IgG on the mouse ERG. To inactivate the IgG, it was denatured by boiling at 98°C for 45 minutes. In order to facilitate transport across the inner limiting membrane, MAR IgG (10 mg/ml in PBS) was mixed with a peptide delivery agent, Chariot (Actif Motif, Carlsbad, CA), at volume ratio of 1∶1.5 just before injection, similar to the method previously described for the delivery of Kir2.1 antibodies to the retina [24]. A 34 gauge needle attached to a Nanofil syringe (World Precision Instruments, Sarasota, FL) pierced the sclera 0.5 mm posterior to the limbus. The needle was then inserted at a 45° angle, 1 mm into the vitreous of an anesthetized mouse (i.p. 50 mg/kg ketamine and 5 mg/kg xylazine). Total injected volume was 2∼4 µl.

### Electroretinography

Mice were dark-adapted overnight (>12 hours), prepared and anesthetized under dim red light as previously described [13], [25]. Full field scotopic ERGs were recorded to a series of flashes ranging between 10^−5^ and 1.4×10^2^ cd-s/m^2^. ERGs were the averages of 5 to 25 responses for dim intensities below 10^−1^ cd-s/m^2^. For intensities above 10^−1^ cd-s/m^2^, three trials were averaged. For brighter stimuli (above 10^0^ cd-s/m^2^), the ERG was recorded to a single flash. The interflash intervals were 5 s for the 10^−5^–10^−1^ cd-s/m^2^ flashes, 15 s for the 5×10^−1^ and 10^0^ cd-s/m^2^ flashes, and 30, 60 and 120 s after the 1, 10 and 100 cd-s/m^2^ flashes, respectively. Scotopic ERGs were amplified (Grass Instruments, West Warwick, RI) at a gain of 2000, and band-pass filtered (0.1 to 1 k Hz). Data were acquired with a National Instruments data acquisition board (sampling rate: 10 kHz; National Instruments, Austin, TX). Traces were recorded with customized software (ERGTool, Dr Richard Weleber, Casey Eye Institute, Portland, OR).

### ERG Data analysis

To determine a-wave and b-wave amplitudes, ERG data were processed and analyzed using MATLAB software (version R2006a; MathWorks). ERG a-wave amplitude was calculated from baseline average voltage over the 10 ms before the flash, to the peak of the negative going response between 5 and 30 ms after the flash. Oscillatory potentials were removed from the ERG using an anticausal low-pass filter (-3dB at 60 Hz; implemented using Matlab's filtfilt function). ERG b-wave amplitude (the peak of the positive going response between 25 to 150 ms after the flash) was measured from baseline or from the a-wave trough if present. The plot of a-wave amplitude against flash intensity was well described by the Naka-Rushton equation:
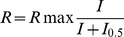
Where Rmax (µV) is the maximal ERG a-wave amplitude and I_0.5_ (cd-s/m^2^) is the flash intensity that produces half the maximal response. The plots of b-wave amplitude versus the light intensity (below 0.1 cd-s/m^2^) were also fit with a Naka-Rushton function. Flashes intensities above 0.1 cd-s/m^2^ were excluded from the analysis of b-wave due to intrusion of cone photoreceptors in the mouse ERG at these higher intensities [26]. Non-linear curve fitting (Origin, OriginLab; Northampton, MA) was used to fit the Naka-Rushton functions to the plots of ERG amplitude against flash intensity.

### Antibody labeling of live, dissociated bipolar cells

Dissociated retinal cells were prepared using the Worthington Papain Dissociation System (Worthington Biochemical Corporation, Lakewood, NJ) as previously described [27]. The cells were dropped onto poly-L-lysine-coated coverslips in 0.5 ml DMEM containing 10% fetal bovine serum, 100 U/mL penicillin, and 100 µg/mL streptomycin in a 24-well tissue culture plate. The cells recovered and attached to the coverslips for 4 hr under 5% CO_2_ at 37°C. MAR serum was added at a dilution of 1∶1000 after which cells were incubated for either 4 hrs or overnight. Cells were then washed twice in cold phosphate-buffered saline (PBS), and fixed for 1–2 min in cold 4% (w/v) paraformaldehyde in phosphate buffer, followed by washing in cold PBS. Finally, fixed cells were placed for an hour at room temperature in Antibody Incubation Solution (3% normal horse serum, 0.5% Triton X-100, 0.025% NaN_3_ in PBS) with mouse anti-PKC alpha (1∶10,000; Novus Biologicals, Littleton, CO), Alexa 488-anti-mouse IgG, and Alexa 594-anti-human IgG (both at 1∶2000; Invitrogen). Coverslips were then washed and mounted onto glass slides for confocal microscopy.

## Results

### Immunoeactivity of MAR sera with TRPM1

**Figure 1:**
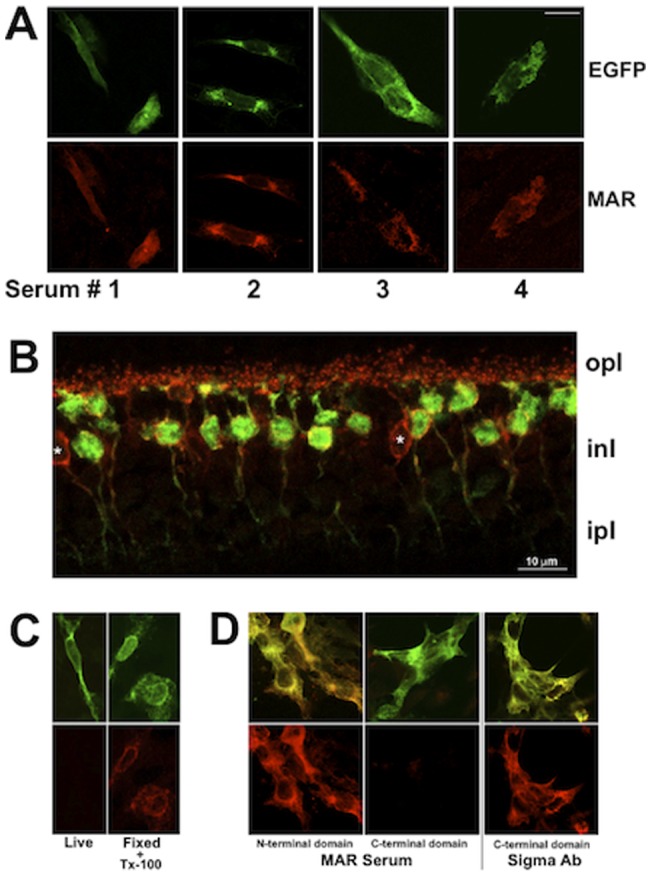
Identification of TRPM1-immunoreactive MAR sera. **A**) MAR sera labeling of CHO cells transfected with EGFP-mTRPM1. EGFP is shown in green and the MAR serum immunoreactivity in red. **B**) Double immunofluorescence labeling of a mouse retina section with MAR serum #2 (red) and anti-PKC(green). Putative cone ON-bipolar cells are marked with asterisks. Scale bars  = 10 µm. Abbreviations: OPL, outer plexiform layer; INL, inner nuclear layer; IPL inner plexiform layer. **C**) CHO cells transfected with EGFP-mTRPM1 were not immunolabeled when incubated with TRPM1-positive MAR serum while alive (left panels). TRPM1 immunofluorescence is revealed by applying the MAR serum after fixing and permeabilizing the cells (right panels). Top images: EGFP, bottom images: MAR immunofluorescence. **D**) Immunofluorescent labeling of N- and C-terminal TRPM1 peptides with MAR serum #2 (left) or a commercial anti-TRPM1 antibody against the C-terminus (Sigma Ab) as positive control (right). Top row: Superimposition of EGFP (green) and immunofluorescence (red) with co-localization appearing yellow. Bottom row: TRPM1 immunofluorescence.

Serum samples from four MAR patients displayed positive immunoreactivity with TRPM1 by immunofluorescent labeling of CHO cells transfected with either GFP-tagged human TRPM1 (EGFP-huTRPM1) or GFP-tagged mouse TRPM1 (EGFP-mTRPM1, Fig. 1A). The cells were fixed, permeabilized and incubated with a range of dilutions of MAR sera (1∶10 to 1∶1000). Immunoreactivity to the sera was visualized with anti-human IgG coupled to a red fluorophore. All four sera selectively labeled cells containing GFP fluorescence for transfections with either EGFP-huTRPM1 or EGFP-mTRPM1 plasmids (i.e. transfected but not non-transfected cells were labeled by the MAR sera). The staining intensity of the four serum samples within the transfected cells varied. One patient sample showed intense immunofluoresence at all dilutions tested. By comparison, the three other samples showed labeling of varying intensities at 1∶100 and no labeling at 1∶1000. In wild-type mouse retinal sections, all four samples showed immunofluorescent labeling of puncta in the outer plexiform layer (OPL); bipolar cell bodies in the inner nuclear layer (INL) could also sometimes be discerned. No immunofluorescent labeling was observed in the OPL of TRPM1−/− retina sections (not shown). The majority of labeled cells were identified as rod bipolar cells by double labeling for PKC [28] (Fig. 1B). A few PKC-negative, seropositive cells were also observed, and based on their morphology, these are most likely cone ON-bipolar cells (Fig. 1B, asterisks). The staining pattern was similar to that observed with a commercial antibody against TRPM1 [13]. These results confirm that TRPM1-positive MAR IgG from the four patients all label retinal ON-bipolar cells, albeit to a varying degree.

### The MAR epitope is located in an N-terminal, cytoplasmic domain of TRPM1

TRPM1 is an integral membrane protein of 1622 amino acids with six predicted transmembrane spans, and intracellular N- and C-terminal domains (Accession: NP 001034193.2). The only possible extracellular epitopes occur in the short loops connecting transmembrane spans 1–2, 3–4 and 5–6, while the bulk of the polypeptide is intracellular. By comparing immunolabeling of permeabilized and non-permeabilized TRPM1-transfected CHO cells (Fig. 1C) and mouse retina sections (not shown), we established that the TRPM1 antigenic epitope was intracellular as suggested previously [22]. To localize the epitope further, plasmids were constructed encoding EGFP fused to either the N-terminal cytoplasmic domain (EGFP-TRPM1-N) or the C-terminal cytoplasmic domain (EGFP-TRPM1-C) from mouse TRPM1. All four MAR sera labeled CHO cells expressing EGFP-TRPM1-N, but not EGFP-TRPM1-C (Fig. 1D). As control, a commercial TRPM1 antibody directed against a human TRPM1 C-terminal polypeptide (aa 1449-1567, UniProtKB/Swiss-Prot: Q7Z4N2) labeled CHO cells expressing EGFP-TRPM1-C, but not EGFP-TRPM1-N (Fig. 1D). These results indicate that the four TRPM1-positive MAR sera react with an epitope within the N-terminal, cytoplasmic domain of the channel.

### MAR autoantibodies are selectively accumulated by live bipolar cells

**Figure 2:**
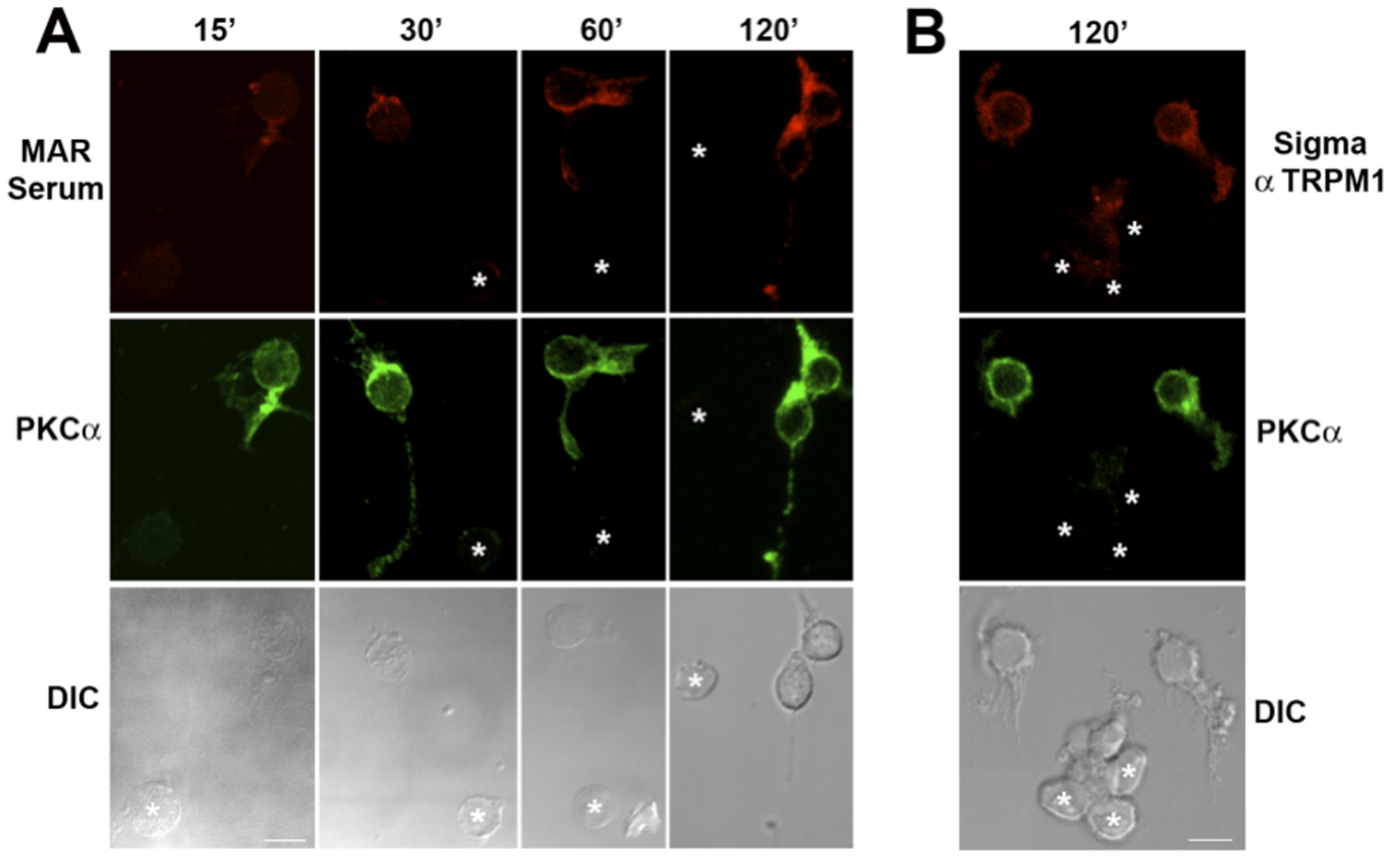
Live rod bipolar cells take up and retain MAR antibodies. Acute cultures of mouse retinal neurons were incubated with MAR serum for 15, 30, 60, or 120 min at 37°C (**A**), or with a commercial affinity purified TRPM1 antibody (Sigma Ab) for 120 min (**B**), then fixed, permeabilized, and the MAR and TRPM1 immunoreactivity visualized with secondary antibodies coupled to Alexa 594 (red, upper row). Following fixation, the retinal cultures were also labeled with a PKC antibody plus anti-mouse Alexa 488 (green, middle row) to identify rod bipolar cells. DIC images (bottom row) show the presence of cells that were negative for both MAR serum and anti-PKCα and which are indicated with asterisks in the upper two rows. Scale bar  = 5 µm.

**Figure 3:**
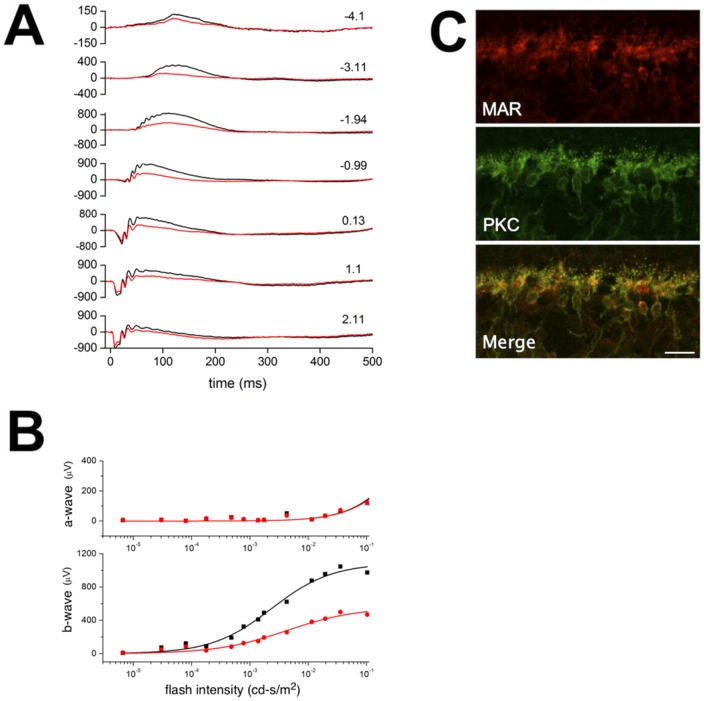
MAR IgG reduces the amplitude of the b-wave originating from the rod bipolar cells. **A**) Representative scotopic ERGs from one of 13 recorded mice. Light flashes were delivered at time 0; numbers to the right indicate scotopic flash intensity (log cd-s/m^2^). Traces from the IgG-injected eye are shown in red and from the sham-injected control eye in black. **B**) Solid lines show the fits of the Naka-Rushton function (eqn 1 in main text) to the plot of scotopic a-wave (top) and b-wave (bottom) peak amplitudes versus flash intensity measured from the above mouse **A** (red: MAR IgG-injected eye; black: control eye). The bottom plot is fitted with the ON-bipolar cell responses to dim light (from 10^−5^ to 10^−1^ cd-s/m^2^). The a-waves of the two traces superimpose, but the b-waves of the MAR IgG-injected eye are of smaller amplitude. **C**) The suppression of the ERG correlates with uptake of the MAR IgG into ON-bipolar cells. After ERG recordings, the retinas were fixed, cryosectioned and reacted with anti-human IgG-Alexa594 (red) to visualize cells that accumulated MAR IgG. The sections were double labeled with PKC (green) to identify rod bipolar cells. The scale bar represents 20 µm.

The intracellular location of the MAR autoantibodies epitope raises the question of how the MAR autoantibodies are able to cross the plasma membrane to bind TRPM1 in living cells. Thus, we examined the uptake of unconjugated MAR autoantibodies into live bipolar cells by culturing dissociated living retinal neurons in the presence of MAR serum #2 (diluted 1∶1000) for 15, 30, 60, and 120 min at 37°C (Fig. 2A). Sequestration of antibodies by retinal cells was assessed at the end of the incubation by fixing and permeabilizing the cells, and then visualizing the MAR IgG with fluorescent anti-human IgG. Fixed cells were double-labeled with an antibody against PKCto identify rod bipolar cells. After 15 min incubation with MAR serum, MAR immunofluorescence was detectable in the dendrites of rod bipolar cells, but not in PKC-negative cells. The intensity of the MAR immunofluoresence in the rod bipolar cells increased with longer incubation times. Immunofluoresence could be observed within cell bodies by 30 min and in the synaptic terminals by 60-120 min. After a two-hour incubation, PKC-positive rod bipolar cells showed stronger MAR immunofluorescence, whereas PKC-negative cells remained unlabeled with the MAR antibodies (Fig. 2A, asterisks). Very rarely, a PKC-negative, MAR positive cell was found, possibly representing a cone ON-bipolar cell. Live, dissociated rod bipolar cells were also able to accumulate a commercial antibody against the intracellular C-terminus of TRPM1 (Fig. 3B), indicating that uptake of antibodies is not limited to MAR serum.

Bipolar cells from wildtype but not TRPM1−/− mice were able to accumulate MAR IgG and the commercial TRPM1 antibody (not shown), indicating that uptake requires TRPM1. However, unlike bipolar cells, TRPM1-expressing CHO cells did not display MAR immunofluorescence following live incubation with the MAR serum. TRPM1 immunoreactivity was only revealed by fixing and permeabilizing the cells prior to incubation with MAR serum (Fig. 1C). These combined results suggest that while the presence of TRPM1 is required for internalization, expression of TRPM1 by a cell does not itself result in internalization of antibodies against TRPM1.

### TRPM1-immunoreactive MAR IgG suppress the ERG b-wave

Lei *et al*. (2000) have previously shown that injection of IgG from MAR patients into the vitreous of the monkey resulted in variable reduction in the ERG b-wave with relative sparing of the a- and d-waves. We sought to confirm and extend these results by investigating whether TRPM1-positive IgG from MAR patients selectively altered the ERG of the mouse. However, preliminary experiments revealed that intravitreal injection of TRPM1-positive MAR IgG did not alter the ERG or result in labeling of the mouse retina, a result also reported by Lei *et al*. (2000) for the rat and guinea pig eye [11]. To overcome possible problems with crossing the inner limiting membrane of the mouse eye, the TRPM1-positivie MAR IgG was mixed with a carrier peptide prior to injection, a method previously validated for the intravitreal delivery of Kir2.1 antibodies to the retina [24]. As a control for non-specific effects of the injection, the same IgG denatured by boiling (heat-inactivated), was similarly mixed with the carrier peptide prior to intravitreal injection of the contralateral eye. Scotopic ERGs were recorded 15-24 hrs after injection. Figure 3A shows representative ERGs from a mouse with one eye injected with active (red trace) or inactivated (black trace) MAR IgG. The ERG b-waves from the eye injected with the active IgG were noticeably reduced. Maximal amplitude of the ERG b-wave was reduced by 47±21.6% (Mean ±SD, n = 13 mice) in the eyes injected with active TRPM1-positive MAR IgG compared with eyes injected with the inactivated IgG (Table 1 and Fig. 3B lower graph). Maximal a-wave amplitude was reduced to a lesser extent than the b-wave with a 20% reduction in eyes injected with TRPM1-positive MAR active IgG (Table 1 and Fig. 3B, upper graph).

**Table 1:** Summary of the ERG results (Mean ± SD, 13 mice) showing changes in the b-wave and a-wave in eyes injected with active MAR IgG or inactivated MAR IgG.

	b-wave without a-wave*		b-wave with a-wave**		a-wave	
	Vmax (µV)	I_0.5_ (cd-s/m^2^)	Vmax (µV)	I_0.5_ (cd-s/m^2^)	Vmax (µV)	I_0.5_ (cd-s/m^2^)
**Active MAR IgG**	295±167	1.2×10^−2^±2.1×10^−2^	546±287	5.7×10^−1^±6.3×10^−1^	456±154	2.2±2.1
**Inactive MAR IgG**	539±190	3.9×10^−3^±2.7×10^−3^	887±272	2.2×10^−1^±9.0×10^−2^	585±186	0.98±0.83
**Statistical Significance**	p<0.05	n.s.	p<0.05	n.s.	n.s.	n.s.

* intensity <10^−1^ cd-s/m2; ** intensity >10^−1^ cd-s/m2; n.s. not significant.

Following ERG recording, the retinas were fixed, cryosectioned, and labeled with a fluorescently tagged secondary antibody to identify cells that had retained the human IgG. Double labeling for PKC identified rod bipolar cells as the primary site for uptake and retention of the injected IgG (Fig. 3C). No bipolar cell labeling was observed for eyes injected with heat-inactivated IgG (not shown). These results for the first time demonstrate the selective uptake and retention of MAR autoantibodies by ON-bipolar cells while also showing a reduction in the amplitude of the ERG b-wave, which is primarily generated by the ON-bipolar cells [29].

## Discussion

The visual symptoms and ERG phenotype characteristic of MAR are similar to those that occur in CSNB1, a congenital visual disease caused by mutations in TRPM1 and other genes required for the ON-bipolar cell light response. Sera from MAR patients have been reported to label retinal bipolar cells, and the ERG b-wave in monkey has been reported to be selectively reduced by intravitreal injection of MAR IgG [11], even though for many years the MAR antigen was unknown. Recently an antigen targeted by MAR autoantibodies was identified as TRPM1, a cation channel normally expressed by both melanocytes and ON-bipolar cells [22], [23]. Here, we have confirmed TRPM1 as a MAR antigen in bipolar cells, by identifying four patient sera that label TRPM1-transfected CHO-K1 cells, as well as retinal bipolar cells in TRPM1+/+ but not TRPM1−/− mouse retina. Furthermore, we show that injection of MAR IgG into mouse eyes leads to attenuation of the ERG b-wave and the appearance of the human antibodies in the retinal ON-bipolar cells. These results demonstrate the ability of MAR autoantibodies to reduce the b-wave amplitude of ERG by targeting a key component of the ON-bipolar cell signal transduction pathway.

In addition to the above work, we further localized the epitope of the TRPM1 autoantibodies to the intracellular, amino-terminal domain by expressing partial TRPM1 sequences. In order to understand how retinopathy may be caused by TRPM1 autoantibodies, retinal neurons were cultured in the presence of MAR serum. We found that MAR autoantibodies are capable of crossing the plasma membrane of live ON-bipolar cells to access the intracellular epitope on TRPM1, resulting in accumulation of MAR IgG in live TRPM1^+/+^ bipolar cells, not TRPM1^−/−^ cells. On the other hand, live CHO-K1 cells expressing EGFP-mTRPM1 did not accumulate MAR autoantibodies following incubation with MAR serum. Together, these results suggest that expression of TRPM1 is necessary, but not sufficient to result in cellular uptake and retention of MAR IgG. The uptake process most likely depends on both a non-specific mechanism for uptake, such as pinocytosis, and TRPM1 expression for retention. Retinal bipolar cells exhibit a high rate of macropinocytosis, an actin-dependent endocytic mechanism for bulk membrane retrieval [30]. In analogy, in cancer-associated retinopathy (CAR), another paraneoplastic autoimmune syndrome in retina, uptake of autoantibodies against intracellular retinal antigens (e.g. recoverin) has been shown to be temperature and actin dependent, consistent with an active endocytic process [31].

In human MAR patients, autoantibodies from the circulating choroidal blood supply must first cross the blood-retinal barrier (BRB), which consists of the RPE and the walls of the retinal vasculature, in order to enter the retina before crossing the ON-bipolar cell membrane [32]. In our experiments, antibodies were introduced by intravitreal injection and must cross the inner limiting membrane (ILM), a basement membrane formed between the endfeet of Müller cells, in order to enter the retina. The permeability of the BRB to antibodies is likely to be quite different from that of the ILM. In mice, intravitreal injection of MAR IgG alone had no effect on the ERG (not shown), consistent with previous results in rats and guinea pigs [11]. However, intravitreal injection of MAR sera in the monkey suppressed ERG b-wave amplitudes, although bipolar cell labeling was not seen at the conclusion of the experiment [11]. The reason for the differences between rodents and monkeys in antibody access to the retina from the vitreous is not yet known. A greater understanding of why TRPM1 autoantibodies do not cross the ILM of the rodent may provide a path to designing treatments for autoimmune and paraneoplastic retinopathies.

To overcome the problems of uptake from the vitreous in mice, we mixed purified MAR IgG with a peptide delivery agent to facilitate transport into the retina, as previously described for potassium channel antibodies [24]. At 24 hours after injection of the mixture of carrier peptide and MAR IgG, ERG recordings showed a marked reduction of the b-wave amplitudes compared with the reduction in the a-wave. After the ERGs, application of fluorescent secondary antibodies revealed the appearance of MAR IgG in ON-bipolar cells. Thus, it is likely that inactivation of TRPM1 channels in ON-bipolar cells by MAR autoantibodies accounts for the suppression of the ERG b-wave in MAR [11]. Though the incidence of clinically diagnosed MAR is infrequent, recent studies have found that the occurrence of anti-retinal autoantibodies in the serum of melanoma patients is more common than previously suspected [33]–[35]. Further screening of serum from melanoma patients will be necessary to determine the prevalence of TRPM1 as a melanoma-associated antigen.
